# Toxicity of λ-cyhalothrin and fenpyroximate on *Nannotrigona testaceicornis*

**DOI:** 10.1007/s11356-025-37169-7

**Published:** 2025-11-20

**Authors:** Jaíne Santos Rebouças, Jefferson Alves dos Santos, Maiara Janine Machado Caldas, Maria Angélica Pereira de Carvalho Costa, Ana Tereza Bittencourt Guimarães, Nuno G. C. Ferreira, Carlos Alfredo Lopes de Carvalho

**Affiliations:** 1 Grupo de Pesquisa Insecta, Universidade Federal Do Recôncavo da Bahia, 44380-000 Cruz das Almas, Bahia Brazil; 2 Ecotoxicology and Landscape Research Group, Rua Universitária N. 2069, Cascavel-PR, Brazil; 3 Faculdades Pequeno Príncipe, Av. Iguaçu, 333, Curitiba-PR, 80230-020 Brazil; 4 CIIMAR/CIMAR-LA, Centro Interdisciplinar de Investigação Marinha e Ambiental, Universidade do Porto, Terminal de Cruzeiros do Porto de Leixões, 4450-208 Matosinhos, Portugal; 5 School of Biosciences, Cardiff University, Museum Avenue, Cardiff, Wales, CF10 3AX United Kingdom

**Keywords:** Pollinators, Meliponini, Ecotoxicology, λ-cyhalothrin; Fenpyroximate

## Abstract

The use of pesticides in agriculture is linked to pollinators’ decline, which also impacts crop productivity. Determining pesticide effects on pollinating bees is of utmost importance for defining crop management strategies and public policies. This study assessed the toxicity of λ-cyhalothrin and fenpyroximate in *Nannotrigona testaceicornis* bees through three exposure routes (ingestion, topical and contact). The concentrations were selected based on field application doses and median lethal concentration for social bee species. Survival and behavioural changes were assessed up to 96 h of exposure. Data showed that λ-cyhalothrin caused a significant decrease in bee survival in all exposure routes, while fenpyroximate did not cause any lethal effect. The Bees’ Behavioural Stress Index (BSI) was created as a simple tool to assess behavioural changes, allowing for the identification of stress levels and significant differences from control groups. The data presented here can be used for protecting stingless bees and as a standard testing method.

## Introduction

The agricultural sector is a critical global activity, with approximately 37.7% of the world’s land surface devoted to food production (Talaviya et al. 2020). Agriculture is essential for sustaining human life providing employment and income (Talaviya et al. 2020). However, the growing demand for food has led to increased pesticide use to control unwanted organisms (Serrão et al. 2022), which has caused significant damage to biodiversity, particularly pollinators (Di Bartolomeis et al. 2019).

Stingless bees (Meliponini) are crucial pollinators responsible for pollinating both native and cultivated plant species across tropical and subtropical regions globally (Matos et al. 2021). Stingless bees comprise over 600 species across 45 genera worldwide (Engel et al. 2023). In Brazil alone, 251 species within 34 genera have been identified (Camargo et al. 2023), including 40 species from 14 genera (Rodrigues et al. 2025).

The stingless bee species *Nannotrigona testaceicornis* (Lepeletier 1836 - Hymenoptera: Apidae) has a broad geographic distribution across Brazil, inhabiting the states of Bahia, Espírito Santo, Goiás, Mato Grosso do Sul, Minas Gerais, Paraná, Rio Grande do Sul, Rio de Janeiro, Santa Catarina, and São Paulo (Camargo et al. 2023). This species is considered a potential pollinator for various plant species, including economically significant crops like mango (*Mangifera indica*) and melon (*Cucumis melo*) that are exported from Brazil (Giannini et al. 2015). In fact, the cultivation of these two crops is economically vital in major agricultural areas like the São Francisco Valley, a globally renowned fruit-producing region (Mesquita et al. 2024; Sampaio Neto et al. 2021). Still, the large-scale production of these fruit trees relies on conventional pest control methods involving pesticide use (Gaboardi et al. 2023).

The insecticide λ-cyhalothrin and the acaricide fenpyroximate are two commonly used pesticides for controlling pests in the previously mentioned crops (MAPA 2024). However, during and after pesticide application in the field, bees face exposure risks through various contamination pathways, which can disrupt their pollination efficiency and endanger the health of their colonies (Zioga et al. 2023).

Pesticide exposure can then have devastating effects on bees, leading to immediate mortality or sublethal effects (Toledo-Hernández et al. 2022). Sublethal effects may manifest in various ways, such as impaired locomotion, tremors, paralysis and memory deficits in adult bees contaminated in the field (Leite et al. 2022). The severity of these effects depends on the mode, duration, and concentration of pesticide exposure (Toledo-Hernández et al. 2022).

The OECD guidelines for bee studies, OECD #213 (OECD 1998a) and OECD #214 (OECD 1998b), require reporting any observed abnormal behaviours during exposure. However, this narrow focus may not fully capture bees’ complex behavioural responses, as no further statistical analyses are reported. Developing a standardised index to evaluate a broader range of behaviours, such as agitation, disorientation, paralysis, prostration, difficulty moving, wing fluttering and self-cleaning, would provide a more comprehensive assessment of bee health and help identify subtle sublethal effects of pesticides and other stressors.

Given the economic importance of the São Francisco Valley region to Brazil and the potential risks that the pesticides λ-cyhalothrin and fenpyroximate pose to stingless bees like *N. testaceicornis*, it is crucial to assess the impacts of these pesticides. This study aimed to evaluate the toxicity of these two pesticides through ingestion, topical application, and surface contact exposure in the stingless bee species *N. testaceicornis*. The doses tested were those recommended for pest control in mango and melon crops using parameters such as mortality and behaviour. This study introduces the bee’s behavioural stress index (BSI) for the first time, which can be used in further studies.

## Material and methods

### Bee sampling and experimental design

Permission to collect and perform the ecotoxicological exposures were obtained from the Biodiversity Authorisation and Information System (SISBIO) under licence number 84168-1. *Nannotrigona testaceicornis* worker bees were collected from 16 standardised colonies (INPA type: 12 cm x 12 cm x 5 cm) in the meliponary located in the municipality of Cruz das Almas, Bahia (12º39’20” W - 39º07’23” S, altitude 220 m). The collections were made in the morning, between 8:30 am and 11:00 am, during the greatest foraging activity, and were always carried out independently on the days prior to the experimental tests to minimise stress on the bees. The colony entrances were sealed with wax, and the foragers were allowed to return (10 to 15 minutes). As the bees returned, they clustered at the entrance of the colony, enabling their capture by using a 500 mL plastic container. Only healthy colonies were selected for the procedure, identified by the absence of visible signs of parasitism and the presence of brood cells with intact envelopes at different stages of development (early and late).

Immediately after collection, the specimens were transferred to the Laboratório de Criação e Comportamento dos Insetos e Ácaros do Grupo de Pesquisa Insecta, Centro de Ciências Agrárias, Ambientais e Biológicas da Universidade Federal do Recôncavo da Bahia (UFRB), Cruz das Almas, Brazil, where they were screened to assess their behavioural condition. Bees showing signs of physiological impairment, such as lethargy or a moribund state, were not used. Following the screening, the healthy bees (n= 1173) were anaesthetised by cooling to −14 ± 2 °C for approximately 1 minute and 25 seconds, then transferred to adapted 500 mL experimental cages (Botina et al. 2020).

Each treatment had three replicates (experimental cages), with 10 individuals in each cage. Bees within each cage originated from the same colony, and each replicate corresponded to a different colony, ensuring that three distinct colonies were used in each test. The bees were kept in Biochemical Oxygen Demand (BOD) incubators, following the procedures described by Botina et al. (2020) and OECD protocols #213 (OECD 1998a) and #214 (OECD 1998b), with adaptations to maintain the temperature at 28 ± 2 °C and humidity at 70 ± 5% to comply with the species’ needs.

### Concentrations and solution preparation

The bioassay concentrations were based on the active ingredient (a.i.) levels of the pesticides λ-cyhalothrin and fenpyroximate in commercial pesticide products recommended for mango and melon cultivation. The lower recommended field application dose (RFAD) for λ-cyhalothrin was based on that defined for the pesticides Kasio 250 CS® and Davos ® applied to mango crops, while the highest RFAD was established according to the pesticide Trinca Caps ® used in melon cultures. In the case of fenpyroximate, the lower and higher RFAD was derived from the pesticides Ortus 50 SC®, Fujimite 50 SC® and Portal® applied to mango crops. These recommendations were obtained from the Brazilian Phytosanitary Agrochemicals System (Agrofit – Ministério da Agricultura e Pecuaria: https://agrofit.agricultura.gov.br/ - MAPA 2024). However, only recommendations for mango were available for fenpyroximate.

The lowest and highest active ingredient (a.i.) concentrations used were calculated from the manufacturers’ guidelines for the recommended field doses for these crops. In addition, preliminary studies using the median lethal concentration (LC_50_) values for social bee species were also used to determine the selected exposure routes (dos Santos 2024). Bees were exposed to the pesticides via three distinct contamination routes: ingestion through food (λ-cyhalothrin: 1.44 - 83.30 μg/mL; fenpyroximate: 12.5 - 50.00 μg/mL), topical application to the body surface (λ-cyhalothrin: 0.01 - 1.38 μg/μL; fenpyroximate: 0.025 - 0.100 μg/μL), and contact with treated surfaces (λ-cyhalothrin: 41.65 - 166.60 μg/mL; fenpyroximate: 18.87 - 100.00 μg/mL). A more detailed list of concentrations is provided in Table 1.

The active ingredients were technical grade (purity ≥95%) λ-cyhalothrin (CAS No.: 91465-08-6) and fenpyroximate (CAS No.: 134098-61-6), purchased from Sigma-Aldrich®.

For the ingestion exposure route, the active ingredients were diluted in solvent. Absolute ethyl alcohol (99.5%) was used as a solvent for λ-cyhalothrin, and acetone (99.5%) was used as a solvent for fenpyroximate. The active ingredient was first diluted in a solvent, and then a 1:1 sucrose solution (sugar to distilled water, m/v) was added to prepare the highest concentration for the experiment (stock solution). From this stock solution, serial dilutions were made using the sucrose solution to obtain the desired concentrations. The topical and contact surface exposure routes followed the same process, but only distilled water was used instead of the sucrose solution. For the insecticide λ-cyhalothrin, the solvent concentrations were 0.67% for ingestion, 20% for topical application, and 0.67% for contact surfaces. The acaricide fenpyroximate had solvent concentrations of 0.4% for ingestion, 20% for topical, and 0.5% for contact surfaces. The solutions of each active ingredient were prepared individually for each route of exposure.

**Table 1:** The concentrations of λ-cyhalothrin or fenpyroximate used for the three exposure routes (ingestion, topical and surface contact). The lowest and highest doses recommended for field application were obtained from Agrofit (https://agrofit.agricultura.gov.br/—MAPA 2024). The estimated LC_50_ values ​​were obtained through preliminary tests with social bee species (dos Santos, 2024) or from the study of Pilling and Jepson (1993). RAFD denotes recommended field application dose

Active Ingredient	Exposure route	Concentration	
λ-cyhalothrin	Ingestion (μg/mL)	1.440	½ LC_50_ *Melipona quadrifasciata anthidioides* (dos Santos, 2024)
		2.880	LC_50_ *Melipona quadrifasciata anthidioides* (dos Santos, 2024)
		5.760	2 × LC_50_ *Melipona quadrifasciata anthidioides* (dos Santos, 2024)
		10.000	Lower RAFD (mango cultures)
		83.300	Higher RAFD (melon cultures)
	Topical (μg/μL)	0.010	Lower RAFD (mango cultures)
		0.083	Higher RAFD (melon cultures)
		0.345	5 × LC_50_ *Apis mellifera* (Pilling and Jepson, 1993)
		0.690	10 × LC_50_ *Apis mellifera* (Pilling and Jepson, 1993)
		1.380	20 × LC_50_ *Apis mellifera* (Pilling and Jepson, 1993)
	Contact surface (μg/mL)	41.650	½ Higher RAFD (melon cultures)
		83.300	Higher RAFD (melon cultures)
		124.950	1.5 × Higher RAFD (melon cultures)
		166.600	2 × Higher RAFD (melon cultures)
fenpyroximate	Ingestion (μg/mL)	12.500	¼ Higher RAFD (mango cultures)
		25.000	½ Higher RAFD (mango cultures)
		37.500	Lower RAFD (mango cultures)
		50.000	Higher RAFD (mango cultures)
	Topical (μg/μL)	0.025	½ Higher RAFD (mango cultures)
		0.037	Lower RAFD (mango cultures)
		0.050	Higher RAFD (mango cultures)
		0.100	2 × Higher RAFD (mango cultures)
	Contact surface (μg/mL)	18.870	½ Lower RAFD (mango cultures)
		37.750	Lower RAFD (mango cultures)
		50.000	Higher RAFD (mango cultures)
		73.500	~ 1.5 × Higher RAFD (mango cultures)
		100.000	2 × Higher RAFD (mango cultures)

### Bioassay via ingestion

Bees were kept in experimental cages (500 mL) for two hours without food or water. The cages were kept in an incubator at 28 °C ± 2 °C and 70% ± 5% relative humidity in the dark. Afterwards, each cage was provided with a sucrose solution (1:1 sugar to distilled water, (m/v)) containing either λ-cyhalothrin or fenpyroximate (see Table 1). For the control treatments, distilled water and a solution with the same proportion of solvent as the initial solution was used (OECD 1998b). To supply the sucrose solution, cotton wool was inserted into microtubes to prevent the bees from drowning. In addition, all experimental groups had access to water *ad libitum*. The exposure lasted for 96 hours.

The microtubes containing the sucrose solution were weighed at the evaluation intervals. For each evaluation interval, the need to replenish the sucrose solution was checked, and if there was a need to replenish the sucrose solution, it was weighed before and after replenishment, making it possible to calculate the total food consumption (g/cage), the average consumption per bee (g/bee - OECD 2017), and the average amount of active ingredient consumed per bee in each treatment (µg/bee).

### Topical bioassay

Bees were previously immobilised by cooling to −14°C ± 2 °C for approximately 1 minute and 25 seconds. Then, a 1 μL volume of the λ-cyhalothrin or fenpyroximate solutions was applied to the dorsal region of the thorax using a micropipette (Table 1). For the control treatments, distilled water and a solution with the same proportion of solvent as the initial solution were used (OECD 1998b). Finally, the bees were offered water and a sucrose solution [1:1 sugar and water ratio (m/v)]. The exposure lasted for 96 hours.

### Bioassay via contact surface

A Limatec® spray tower (Vieira et al. 2024) was used to apply 1 mL of a λ-cyhalothrin or fenpyroximate solution to the interior of the experimental cage (Table 1), which had been padded with Whatman® No. 1 filter paper (Del Sarto et al. 2014; Leite et al. 2022). After the experimental cages had dried for 30 minutes at room temperature, the group of 10 bees was carefully transferred individually to the experimental cages that had been previously sprayed and dried. Then, two 1.5 mL microtubes were placed in each cage - one containing cotton wool soaked in water and the other with a 1:1 (m/v) solution of sugar and water. The exposure lasted for 96 hours.

### Bees’s behavioural stress index

In all exposure routes, bees were kept in an incubator at a temperature of 28 °C ± 2 °C and 70% ± 5% relative humidity in the dark. Their survival rate and sublethal effects were evaluated after 1, 6, 12, 24, 48, 72, and 96 hours (Leite et al. 2022). Behavioural observations were conducted (approximately 3–7 min per cage) and documented in real time throughout the experimental process. Bees were considered dead if they did not respond to mechanical stimuli, such as touching their body surface with a soft bristle brush (Del Sarto et al. 2014). Sublethal effects assessed included disorientation, paralysis, prostration, difficulty moving, agitation, wing fluttering, and self-cleaning (Leite et al. 2022; OECD 2017).

To calculate the bee’s behavioural stress index (BSI), the following equation was used:

$$BSI=\left[1-\left(\frac{{\sum }_{0}^{t}DOrg}{100}\right)\right]*\left(\frac{\sum BA}{BC \times 100}\right)+ \left(\frac{DOrg}{100}\right)$$

where: *DOrg* is the percentage of dead organisms at a specific time point (t) since the start of the exposure (time = 0 h); *BA* is the percentage of altered organisms at each behavioural category; *BC* is the number of behavioural categories (agitation, disorientation, paralysis, prostration, difficulty moving, wing fluttering and self-cleaning). If all organisms are dead, BSI score should be considered as 1. A detailed description of the behavioural categories is provided in Table 2.

**Table 2:** Different behavioural types and corresponding descriptions for the different categories of the Bees’ Behavioural Stress Index (BSI)

Behviour Type	Description
Paralysis	Bees are unable to move and exhibit only minimal movements of the legs and antennae, with a weak response to contact stimulation (Lourenço et al. 2012; OECD 2017; Leite et al. 2018)
Difficulty Moving	Bees remain standing and attempt to move, but display reduced coordination and noticeable tremors (OECD 2017)
Agitation	Bees exhibiting rapid movement or flight within the cage in response to the light stimulus (OECD 2017)
Disorientation	Bees that move without difficulty but do not follow other bees heading toward the light by either circle continuously or moving back and forth across the cage (Leite et al. 2018)
Prostration	Bees remain motionless in the cage and exhibit only minimal or delayed responses to contact stimulation (OECD 2017)
Wing fluttering	Bees are either stationary or moving around the cage with their abdomens raised and wings beating rapidly (Lourenço et al. 2012)
Self-cleaning	Bees exhibit excessive self-cleaning behaviour by vigorously using their legs (Leite et al. 2018; OECD 2017)

The *BSI* resulted in a score between 0 and 1, categorised as <0.2 - No Stress; 0.2 - 0.4: Low Stress, 0.4 - 0.6: Moderate Stress; 0.6 - 0.8: High Stress and > 0.8: Extreme Stress.

### Statistical analysis

A significance level of α=0.05 was used for all analyses, and the analyses were conducted using R software (R Core Team, 2024).

Kaplan-Meier survival curves were generated to determine the proportion of surviving bees over time after exposure to the different treatments, using the “survfit” function from the Survival package (Therneau 2024). The overall similarity between the survival curves was assessed using the Log-Rank test (Bland and Altman 2004) with the “survdiff” function from the Survival package (Therneau 2024). When the null hypothesis was rejected, pairwise comparisons of the treatments were made using the Log-Rank test and the “pairwise_survdiff” function from the “survminer” package (Kassambara et al. 2024), with *p*-values corrected using the Benjamini-Hochberg method.

For the ingestion exposure route, the total consumption (sum of evaluations) of sucrose diet (g) per bee in each repetition during the 96-hour evaluation period was calculated (OECD 2017). The data was tested for normality (Shapiro-Wilk test) and homogeneity of variances (Bartlett’s test). Assuming these assumptions were met, an Analysis of Variance (ANOVA) was conducted using the “dic” function from the “ExpDes.pt” package (Ferreira et al. 2021), followed by the Scott-Knott post-hoc test to group the means that did not differ statistically. Only data within the standard distribution range was considered for analysis of food consumption through ingestion. This range was defined as values at or below the upper limit (Q3 + 1.5 × interquartile range) and at or above the lower limit (Q1 - 1.5 × interquartile range).

The dose–response analysis calculated the median lethal concentration (LC_50_) for ingestion and topical exposure along with the median lethal dose (LD_50_) and median lethal dose per gram of bee (LD_50_ weight). This analysis took into account bee mortality data over 96 h of exposure. A log-logistic regression model was employed using the ‘drm’ function from the ‘drc’ package (Ritz et al. 2015). Afterwards, the median lethal dose (LD_50_) and median lethal concentration (LC_50_) values from the generated regression, using the ‘ED’ function in the ‘drc’ package (Ritz et al. 2015), were estimated.

Linear mixed models were fitted to analyse the bee’s behavioural stress index (BSI). They considered the treatments with different concentrations of each pesticide (λ-cyhalothrin and fenpyroximate) as a fixed factor across the exposure routes (ingestion, topical, and surface) over 96 h. The models were carried out using the ‘lme4’ package (Bates et al. 2015), and comparisons were made by contrasts using the ‘emmeans’ package (Lenth 2024).

## Results

### λ-cyhalothrin exposure: ingestion route

**Fig. 1:**
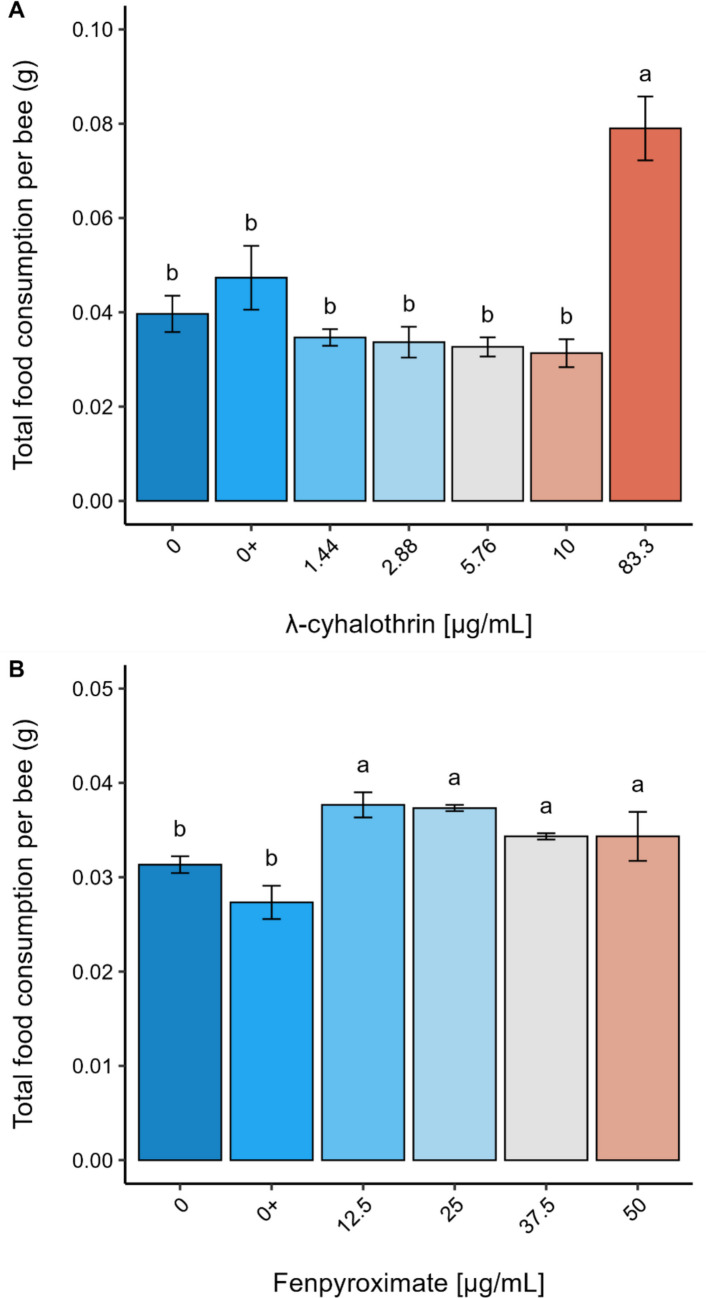
Average ± standard error (SE) of food consumption (g) by *Nannotrigona testaceicornis* after 96 h of exposure to λ-cyhalothrin (**A**) or fenpyroximate (**B**). Different letters correspond to statistically different groups. 0 + denotes control solvent

**Fig. 2:**
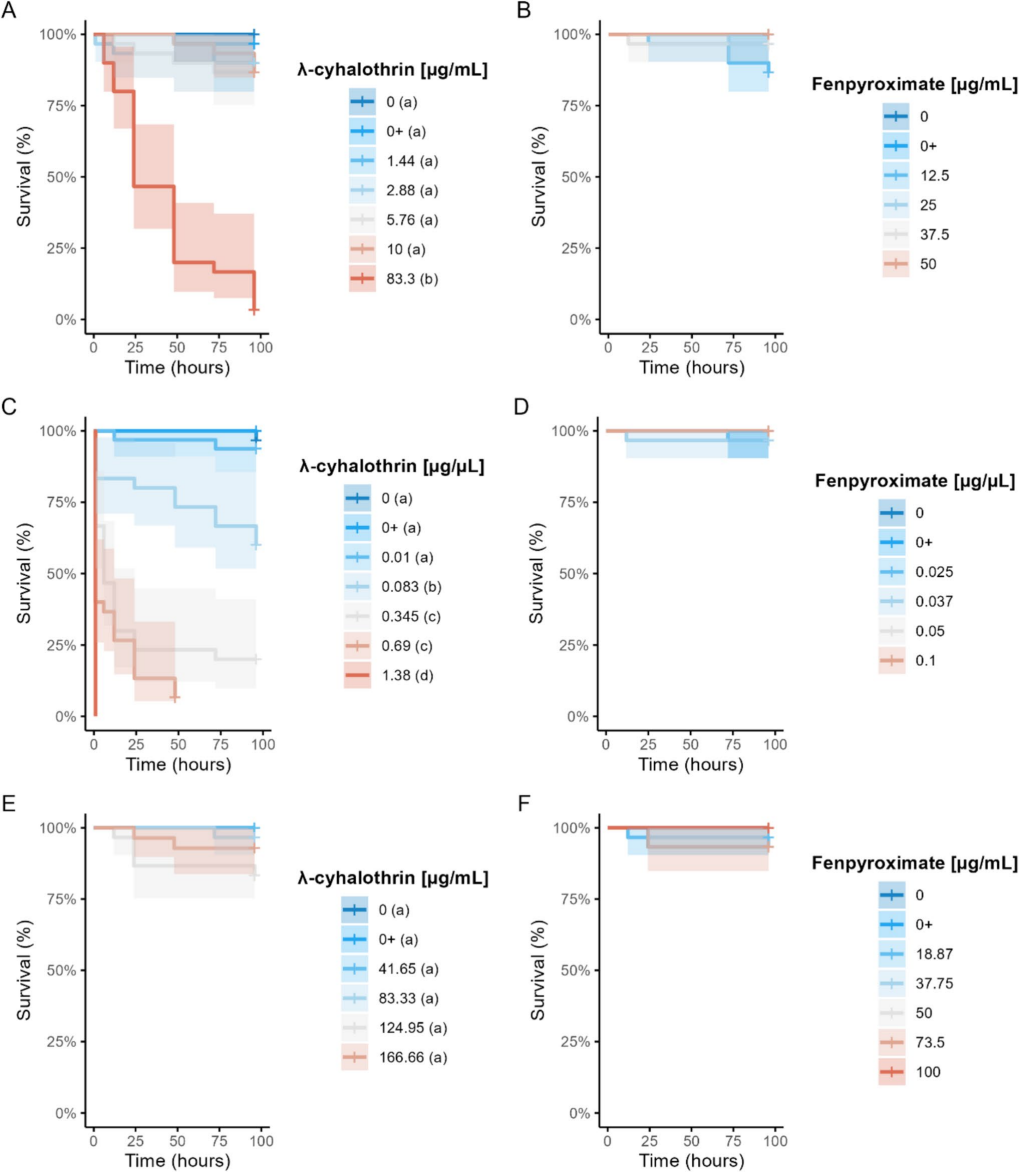
Survival curves and 95% confidence interval for *Nannotrigona testaceicornis* exposed to λ-cyhalothrin or fenpyroximate up to 96 h of exposure through different exposure routes: **A**) λ-cyhalothrin: ingestion route; **B**) λ-cyhalothrin: topical contact route; **C**) λ-cyhalothrin: contact surface route; **D**) fenpyroximate: ingestion route; **E**) fenpyroximate: topical contact route; **F**) fenpyroximate: contact surface route. Different letters accompanying the concentrations in the legend correspond to statistically different groups. 0 + denotes control solvent

The consumption rate showed a significant statistical difference between the exposure groups (F_6,13_ = 13.753; *p* < 0.0001 - Figure 1A). The bees consumed more of the sucrose diet containing the highest concentration of λ-cyhalothrin (83.3 μg/mL). The acute exposure of *N. testaceicornis* bees to the insecticide λ-cyhalothrin significantly decreased their survival rate (Log-Rank test: χ2 = 194, df = 6, *p* ≤ 0.0001). A paired comparison test revealed that the 83.3 μg/mL concentration was statistically different from the other concentrations (*p* < 0.0001), with 3% of bees surviving after 96 hours of exposure. In contrast, the other concentrations - 1.44 μg/mL (90% survival), 2.88 μg/mL (90% survival), 5.76 μg/mL (86% survival), 10 μg/mL (86% survival), control (100% survival), and solvent control (96% survival) - did not differ statistically from each other (*p* > 0.05; Figure 2A). The LC_50_ and LD_50_ values were estimated whenever possible (Table 3).

**Table 3:** Medium lethal concentration (LC_50_) or medium lethal dose (LD_50_) and correspondent 95% confidence interval (C.I.) for *Nannotrigona testaceicornis* exposed for different time periods (24 h to 96 h) to λ-cyhalothrin through ingestion or topical exposure routes

λ-cyhalothrin							
Exposure route	Ingestion ^a,b^			Topical ^c,d,e^			
Time	48 h	72 h	96 h	24 h	48 h	72 h	96 h
LC_50_ [95% C.I.]	35.22 [18.90–51.55]	29.71 [15.07–44.35]	18.92 [11.32–26.52]	0.17 [0.12–0.23]	0.14 [0.09–0.18]	0.11 [0.07–0.15]	0.10 [0.06–0.14]
LD_50_ [95% C.I.]	0.70 [0.33 −1.08]	0.85 [0.35–1.35]	0.63 [0.32–0.93]				
LD_50_ weight [95% C.I.]	-	-	-	25.41 [17.02—33.81]	20.36 [13.85—26.86]	16.22 [10.45—21.99]	15.33 [9.38—21.28]

^**a**^ LC_50_: μg/mL; LD_50_: μg/bee. ^**b**^ LD_50_ was calculated by the average of food consumed in each treatment at each time point and the concentration present in food. ^**c**^ LC_50_: μg/μL; LD_50_: μg/bee; LD_50_ weight: μg/g bee. ^**d**^ LD_50_ was calculated by multiplying LC_50_ value by the volume of pesticide solution deposited on each worker bee (1 μl/bee). ^**e**^ LD_50_ weight is the lethal dose (μg) per gram of bee weight, calculated by dividing LD_50_ (μg/bee) by the average weight of bees (0.00677 g/bee)

**Fig. 3:**
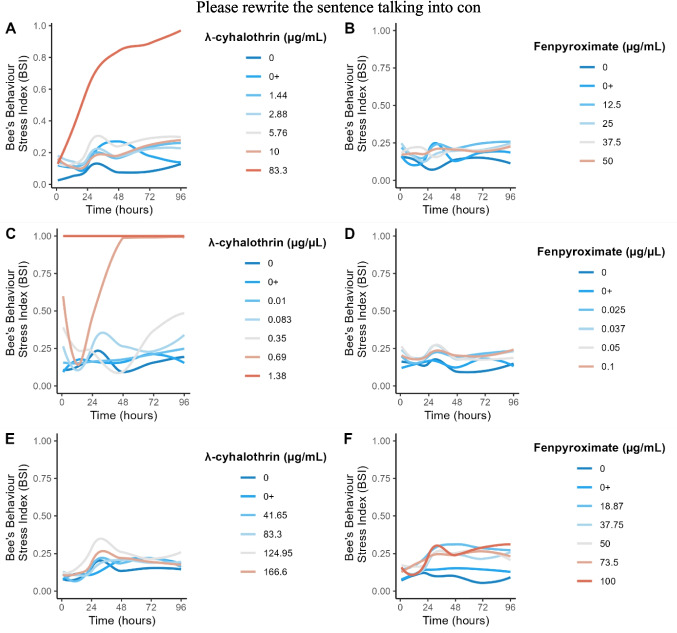
Mixed linear models for the bee’s behavioural stress index (BSI) determined for *Nannotrigona testaceicornis* exposed to λ-cyhalothrin or fenpyroximate up to 96 h of exposure through different exposure routes: **A**) λ-cyhalothrin: ingestion route; **B**) λ-cyhalothrin: topical contact route; **C**) λ-cyhalothrin: contact surface route; **D**) fenpyroximate: ingestion route; **E**) fenpyroximate: topical contact route; **F**) fenpyroximate: contact surface route. 0 + denotes control solvent

During exposure, surviving bees exhibited sublethal effects such as agitation, constant wing flapping, disorientation, difficulty moving, and paralysis. The BSI generally indicated no stress or low stress levels during the initial 24-hour acclimation period. However, the highest concentration (83.3 μg/mL) showed a different response - after 6 hours, it entered a low stress level, then progressed to moderate stress at 12 hours, high stress at 24 hours, and extreme stress levels after that. The behavioural analysis revealed significant differences between groups (LRT = 201.94; *p* < 0.0001). Bees exposed to 83.3 μg/mL of λ-cyhalothrin had a significantly higher BSI than the control and solvent control after 12 hours of exposure (*p* = 0.0069 and *p* = 0.0365, respectively). This treatment (83.3 μg/ml) also had a significantly higher BSI than all other groups after 24 hours (*p* < 0.05; Figure 3A).

### λ-cyhalothrin exposure: topical route

The different concentrations had a statistically significant impact on the survival of *N. testaceicornis* bees (χ2 = 207, df = 6, *p* ≤ 0.0001). Paired comparisons of the survival curves revealed that the 1.38 μg/μL concentration was the most detrimental, causing 100% mortality within 1 hour of exposure (*p* < 0.001). After 96 hours, survival rates were 93% (0.1 μg/μL), 60% (0.083 μg/μL), 20% (0.345 μg/μL), and 6% (0.69 μg/μL), while the control and solvent control had 96% and 100% survival rates, respectively (Figure 2B).

Bees exposed through the topical route exhibited significantly lower survival rates compared to those exposed through other routes. The LC_50_ and LD_50_ values were estimated and presented in Table 3 whenever possible.

The surviving bees displayed behavioural alterations throughout exposure (LRT = 127,26; p < 0,0001). Bees exposed topically to 0.690 μg/μL of λ-cyhalothrin exhibited significantly higher BSI when compared to other groups during the first hour and throughout the experimental period (*p* < 0.05; Fig. 3B). The BSI initially displayed a fluctuation period during the first 12 h. However, after 24 h of exposure, the 0.690 μg/μL treatment rapidly progressed from moderate to extreme stress levels in the subsequent hours. The 0.345 μg/μL treatment displayed moderate stress levels after 72 and 96 h of exposure. As expected, a BSI score of 1 was observed for the highest treatment (1.38 μg/μL) due to its mortality rate.

### λ-cyhalothrin exposure: contact surface route

The survival rate of *N. testaceicornis* bees after 96 hours of exposure was 100% (41.65 μg/mL), 96% (83.3 μg/mL), 83% (124.95 μg/mL), and 92% (166.6 μg/mL), compared to 100% for the control and solvent control groups. Statistical analysis revealed significant differences between the groups (χ2 = 15.6, df = 5, *p* = 0.008). However, pairwise comparisons of the survival curves did not identify any significant differences (*p* > 0.05 - Figure 2C), preventing the calculation of the LC_50_. The surviving bees also exhibited sublethal effects during the exposure period. However, there were no statistically significant differences between the groups over the exposure times LRT = 35.914; *p* = 0.211; Figure 3C).

### Fenpyroximate exposure

The sucrose diet consumption rate differed statistically between the exposure groups (*F*_5, 12_ = 5.9289, *p* = 0.0069), with bees consuming more contaminated diets than the control (Figure 1B). Additionally, no significant differences were observed between the control and the solvent control.

The overall survival curves of the *N. testaceicornis* bees showed no significant differences within the ingestion (χ2 = 9.7, df = 5, *p* = 0.08), topical (χ2 = 3, df = 5, *p* = 0.7), and contact surface (χ2 = 7.7, df = 6, *p* = 0.3) exposure routes. All control groups exhibited survival rates between 96% and 100% (Figure 2D-F).

Despite no high mortality rates were observed across each exposure route, the surviving bees displayed behavioural alterations (e.g., agitation, disorientation, and difficulty moving). While ingestion and topical exposure showed no statistically significant differences between the groups (*p* > 0.05), the contact surface route revealed significant differences between the groups and exposure times (LRT = 61.619; *p* = 0.005). All the experimental groups displayed substantially higher BSI values compared to the control or solvent control groups after 72 h of surface exposure (*p* < 0.05; Fig. 3D-F).

## Discussion

Bees, as key pollinators, have been the focus of numerous studies primarily due to various frameworks aimed at presenting essential policies to reduce their mortality and enhance their ecosystem services. However, most of this research has centred on the honeybee *Apis mellifera*, native to Asia, Africa, the Middle East, and Europe (Carr 2023), overlooking many other essential bee species, particularly on the American continent. To address this gap, the present study examines the potential impact of the insecticide λ-cyhalothrin and the acaricide fenpyroximate on the survival, feeding rates, and behaviour of the stingless bee *N. testaceicornis*. Notably, this study introduces the bee’s behavioural stress index (BSI) as a novel tool to support further research, as numerous studies have already demonstrated that behavioural changes should be considered a key assessment factor (e.g., Bertram et al. 2024).

Pollinators, whose primary dietary sources are pollen and nectar, face contamination risks when pesticides are applied in agricultural settings (Boyle et al. 2019). Exposure can occur through direct contact with treated surfaces or during application when pesticides deposit on foraging bees, as well as through the ingestion of contaminated resources, such as pollen, nectar, or surface water (Boyle et al. 2019). Our study emphasises the critical need to investigate these distinct exposure routes as they determine pesticide uptake dynamics and subsequent toxicity profiles. The pyrethroid insecticide λ-cyhalothrin rapidly disrupts the nervous system due to its lipophilic properties, enabling efficient penetration into cellular membranes and tissues (de Castro et al. 2020). The differences observed across exposure routes reveal significant variations in sensitivity, exemplified by a 6 times higher difference in LD_50_ (96h): 0.63 [0.32 - 0.93] μg/bee for ingestion versus 0.10 [0.06 - 0.14] μg/bee for topical exposure. Ingestion delays systemic distribution due to midgut absorption and potential detoxification pathways, while topical exposure enables rapid cuticle penetration, directly targeting neuromuscular function.

Exposure to pyrethroid pesticides disrupts the kinetics of voltage-dependent sodium channels, prolonging their opening and increasing sodium influx into neurons. This alters the action potential waveform, leading to detrimental effects (Elser et al. 2022). The neurotoxicity of λ-cyhalothrin, a type II pyrethroid, can also affect calcium, chloride, and potassium channels, depending on the cell membrane potential (Elser et al. 2022). These neurotoxic effects can manifest as behavioural changes, loss of motor coordination, paralysis, and mortality (de Castro et al. 2020).

Assessing the ecotoxicological impact of pesticides on bee populations can be a complex process. The diverse exposure pathways, including ingestion, topical absorption, and surface contact, further complicate the assessment process. Moreover, the scientific literature often conflates median lethal concentrations (LC_50_) and median lethal doses (LD_50_), leading to challenges when comparing results across different studies. Consequently, drawing meaningful comparisons between various studies becomes a difficult task. In a previous study, the 96-hour median lethal concentration (LC_50_) for the honey bee (*A. mellifera*) exposed to the insecticide λ-cyhalothrin through contaminated food was 174.4 [146.8–209.5] μg/mL (Wang et al. 2020). Although the study used a commercial formulation (Karate Z 2.08 CS, 22.8% A.I., Syngenta), temperature of 33 ± 0.5 °C, with 65 ± 3% relative humidity, and no light, the LC_50_ value is approximately 10 times higher than the LC_50_ of 18.92 [11.32–26.52] μg/mL found for the stingless bee *N. testaceicornis* (this study).

Notably, the LC_50_ for *N. testaceicornis* falls close to the lowest recommended field application dose of λ-cyhalothrin (10 μg/mL), raising significant concerns about the survival of this species. This concern is further amplified by the 48-hour and 72-hour LC_50_ values for *N. testaceicornis*, which are both below 40 μg/mL, approximately half the higher recommended field application dose (83.3 μg/mL). Even more alarmingly, the stingless bee *Partamona helleri* exhibits an extremely low LC_50_ of 0.043 [0.033–0.055] μg/mL (Motta et al. 2023), indicating a significantly greater threat from exposure to this pesticide. Previous research has demonstrated that acute exposure to λ-cyhalothrin can influence the expression levels of the glutamate receptor A (GluRA) and N-methyl-D-aspartic acid receptor 1 (Nmdar1) genes, which are linked to learning performance and memory in bees (Liao et al. 2018). Such alterations can directly impact foraging activity and communication between bees, thereby jeopardising the transmission of information about food sources (Liao et al. 2018).

The estimated topical LC_50_ values for λ-cyhalothrin are lower than 0.2 μg/μL, which aligns with the 24h-LC_50_ of 0.22 μg/bee and 72h-LC_50_ of 0.11 μg/bee reported for *Bombus terrestris* (Marletto et al. 2003), as well as the 24h-LC_50_ of 0.102 [0.073 - 0.133] μg/bee for *A. mellifera* (Johnson et al. 2006). This is expected, as the insecticide penetrates the insect cuticle more quickly through topical exposure than ingestion (de Castro et al. 2020). However, the lack of published data for other bee species limits further discussion. Notably, the LC_50_ values are slightly higher than the recommended field application dose, highlighting the potential impact of this insecticide on the stingless bee species.

For surface contact exposure, although concentrations up to 166.6 μg/mL were used (twice the higher recommended field application dose), only a maximum of 20% mortality was observed within the 96-hour exposure period. With a 96h-LC_50_ > 166.6 μg/mL, *N. testaceicornis* can be considered less sensitive than *A. mellifera*, which had a 48h-LC_50_ of 131.19 μg/mL (Zhu et al. 2015). However, it is important to note that the exposure in this study involved spraying the surface that the bees contacted rather than directly spraying the bees. This difference in exposure method makes the comparison to *A. mellifera* more challenging, although the surface contact approach may be more representative of realistic field conditions where bees would encounter the applied pesticide.

The United States Environmental Protection Agency has classified λ-cyhalothrin as highly toxic for both ingestion and topical exposure routes (NPIC 2001). This classification is based on the insecticide’s half-life of five days on plant surfaces and its acute oral LD_50_ < 2 μg/bee in the honey bee (*A. mellifera*). Notably, the observed LD_50_ < 2 μg/bee was also observed for oral and topical exposure for *N. testaceicornis*, indicating a high level of toxicity for this species as well.

Even though the previous classification does not consider bee behavioural changes, the results presented in this study could prove very useful and potentially be implemented in the USEPA’s criteria. The BSI results indicate that low stress levels were observed, with no noticeable mortality, particularly in surface contact exposure to λ-cyhalothrin. In contrast, ingestion exposure led to increased BSI stress levels along with higher mortality rates. The importance of using behavioural parameters has been highlighted in prior studies. Current literature suggests that neonicotinoids, a class of insecticides known to have a significant impact on bees, can induce motor function impairment in bees after 24 hours of oral exposure at sublethal doses (10 nM), which is likely to affect normal bee function in field settings (Williamson et al. 2014). The use of behavioural parameters in risk assessment is also evidenced by Thompson (2003), which provides a comprehensive list of pesticide effects under laboratory, semi-field, and field studies, including alterations in labour division, foraging, conditioned responses, effects on colony development, and nestmate recognition, among others.

Fenpyroximate is a widely used acaricide in apicultural practices, primarily employed to control *Varroa* mites in honey bee (*A. mellifera*) colonies. It acts by inhibiting mitochondrial complex I electron transport, disrupting energy production within mite cells (Dahlgren et al. 2012; Johnson et al. 2013). Its broad-spectrum efficacy and targeted activity make it a valuable tool for managing *Varroa* infestations.

Although fenpyroximate is not directly used in meliponiculture (the management of stingless bee colonies), its application in nearby *A. mellifera* colonies, which are commonly managed alongside, can still represent a potential route of exposure for stingless bees. This active ingredient has been used in apiaries in some countries, such as Canada, to control the *Varroa destructor* mite (Bahreini et al. 2022). The safe use of fenpyroximate in *A. mellifera* colonies is also supported by its relatively high 72h-LD_50_ values for ingestion (> 118.5 μg/bee - FAO, 2015) and topical exposure (> 15.8 μg/bee - FAO, 2015). These values are still much higher than the ones reported by USEPA (2020) for the 72 h oral exposure: LC_50_ = 6 μg/g diet; LD_50_ = 0.2 μg/bee and by Dahlgren et al. (2012) for the 48 h topical exposure: LD_50_ = 30 [24.7–36.0] μg/g, making it challenging to compare with *N. testaceicornis* values found on this study (oral: LD_50_ > 1.7 μg/bee; topical: LD_50_ > 0.1 μg/bee). Despite the differences observed across exposure routes, the highest tested concentrations corresponded to 1 or 2 times the recommended field application dose, indicating low toxicity to this species. Similarly, Leite et al. (2018) reported that *Apis mellifera* exposed to the highest recommended field application dose via ingestion and surface contact exhibited approximately 95% and 80% survival rates after 96 hours of exposure.

The low mortality caused by fenpyroximate does not imply that this active ingredient is harmless to pollinators. Investigating the sublethal effects resulting from acute or chronic exposure is necessary. In this study, bees exposed to fenpyroximate at all concentrations and through all three exposure routes exhibited behavioural changes, including constant agitation, disorientation, and locomotion difficulties. In contrast, differences in the bee’s behavioural stress index (BSI) were observed only for the contact surface route when compared to λ-cyhalothrin, revealing significant differences between the treatments and the control group after 72 hours of exposure. These findings align with previous observations of *A. mellifera* exposed to fenpyroximate, which also reported locomotion issues (Leite et al. 2018). Notably, the BSI values in this study remained within the low-stress range and did not reach moderate stress levels.

## Conclusions

This research emphasises the importance of investigating the effects of various pesticides on non-*Apis* bees, particularly stingless bee species. The introduction of the Bee Stress Index (BSI) presents a novel and innovative tool that can complement traditional experimental methods, incorporating behavioural parameters such as individual survival rates and bee behaviour. To our knowledge, this study is the first to examine the impacts of fenpyroximate and λ-cyhalothrin on the survival and behaviour of *N. testaceicornis*. Our findings reinforce the importance of including non-*Apis* species in pesticide risk assessments, particularly in Brazil, given the country’s remarkable diversity of stingless bees and their sensitivity to pesticides.

## Data Availability

Data will be made available at Zenodo - 10.5281/zenodo.14359028.

## Declarations

### Artificial Intelligence (A.I.) statement

During the preparation of this study, the authors used the Copy.ai Paragraph Rewriting Tool to enhance linguistic quality. After using this tool, the authors reviewed and edited the content as needed and assume full responsibility for the content of the published article.
